# Vertically integrated shared learning models in general practice: a qualitative study

**DOI:** 10.1186/1471-2296-14-144

**Published:** 2013-10-01

**Authors:** Christine M Ahern, Thea F van de Mortel, Peter L Silberberg, Janet A Barling, Sabrina W Pit

**Affiliations:** 1 North Coast GP Training, PO Box 1497, Ballina NSW 2478, Australia; 2 School of Health and Human Sciences, Southern Cross University, PO Box 157, Lismore NSW 2480, Australia; 3 University Centre for Rural Health, North Coast, Sydney School of Public Health, The University of Sydney, PO Box 307, Lismore NSW 2480, Australia

**Keywords:** Registrars, Prevocational trainees, Medical students, Medical education, Vertical integration, Near-peer teaching, General practice, Postgraduate training

## Abstract

**Background:**

The numbers of learners seeking placements in general practice is rapidly increasing as an ageing workforce impacts on General Practitioner availability. The traditional master apprentice model that involves one-to-one teaching is therefore leading to supervision capacity constraints. Vertically integrated (VI) models may provide a solution. *Shared learning*, in which multiple levels of learners are taught together in the same session, is one such model. This study explored stakeholders’ perceptions of shared learning in general practices in northern NSW, Australia.

**Methods:**

A qualitative research method, involving individual semi-structured interviews with GP supervisors, GP registrars, Prevocational General Practice Placements Program trainees, medical students and practice managers situated in nine teaching practices, was used to investigate perceptions of shared learning practices. A thematic analysis was conducted on 33 transcripts by three researchers.

**Results:**

Participants perceived many benefits to shared learning including improved collegiality, morale, financial rewards, and better sharing of resources, knowledge and experience. Additional benefits included reduced social and professional isolation, and workload. Perceived risks of shared learning included failure to meet the individual needs of all learners. Shared learning models were considered unsuitable when learners need to: receive remediation, address a specific deficit or immediate learning needs, learn communication or procedural skills, be given personalised feedback or be observed by their supervisor during consultations. Learners’ acceptance of shared learning appeared partially dependent on their supervisors’ small group teaching and facilitation skills.

**Conclusions:**

Shared learning models may partly address supervision capacity constraints in general practice, and bring multiple benefits to the teaching environment that are lacking in the one-to-one model. However, the risks need to be managed appropriately, to ensure learning needs are met for all levels of learners. Supervisors also need to consider that one-to-one teaching may be more suitable in some instances. Policy makers, medical educators and GP training providers need to ensure that quality learning outcomes are achieved for all levels of learners. A mixture of one-to-one and shared learning would address the benefits and downsides of each model thereby maximising learners’ learning outcomes and experiences.

## Background

General Practitioners (GPs) have a pivotal role in delivering primary health care services and training future GPs to do so. However, general practice in Australia is undergoing a period of transformation due to an ageing workforce [1] and a gender shift [2], both of which are associated with GPs working fewer hours. General practice as a place of training is also undergoing transformation. Of most significance is the increased number of medical students (MSs), prevocational trainees (PTs) and GP registrars (GPRs) requiring general practice training. For example, between 2000 and 2010, the number of Australian medical graduates almost doubled and a further 46% increase is projected by 2016 [3]. To facilitate the consequent influx of registrars and prevocational trainees [4] the number of registrar training places will double between 2008 and 2014, and the number of prevocational places is expected to more than double between 2009 and 2013 [5], impacting on training costs, supervision loads, and service capacity [6].^a^ Similar problems are occurring on an international scale [7]. General Practice Education and Training [8] suggests that vertically integrated (VI) teaching models may provide a means of addressing capacity issues. Shared learning, in which multiple levels of learner are taught together in the same session [9], is one such model.

Dornan et al. [10] suggests that the learning process is enhanced by being part of a learning community rather than simply receiving instruction from a teacher. Communities of practice are commonly used in the healthcare sector to generate and share knowledge and improve performance [11]. Wenger et al. [11] define a *community of practice* (CoP) as a ‘learning partnership among people who find it useful to learn from and with each other about a particular domain, [who] use each other’s experiences of practice as a learning resource…and join forces in making sense of and addressing challenges they face individually or collectively’ in a way that influences their practice. The community can be developed formally or informally, and the intention to sustain learning and share knowledge and skills can be tacit or explicit. Based on this description, shared learning models may be a form of community of practice.

Based on small numbers of interviews with a limited range of stakeholders, various benefits and risks of shared learning have been theorised, including a reduction in the workload of the GP teacher [12], improved team morale, patient care and reputation [13]; and the opportunity for learners to acquire survival skills from those ahead of them in the training pathway [14]. While the different curriculum requirements of registrars and students can make the choice of shared topics difficult [12], shared learning can potentially encourage collegiality and improve learning quality.

For example, following a shared learning trial, British GPRs (n=7) [9] suggested they experienced less isolation, enjoyed sharing experiences and the support gained from one another, learnt from the mistakes of others, and appreciated the opportunity to benchmark their progress against their peers. Reduced time with their supervisor, personality clashes between registrars, and receiving non-constructive feedback from peers were reported disadvantages. It was unclear whether the registrars were sharing learning with others at the same or a different level to themselves, and other levels of learner such as prevocational trainees or students did not appear to be involved, so the results cannot necessarily be extrapolated to other settings in which multiple levels of learner are taught together. Other concerns with shared learning include maintaining teaching quality and quantity [13], fears by learners ‘*that their learning objectives may be overwhelmed by the assimilation of other learners into their activities’* ([14] p. 11), and managing variability in prior learning experiences [14].

Given the paucity of learners’ perspectives in the shared learning literature, the study aim was to explore learners’ and other stakeholders’ perceptions of shared learning models in general practice, in order to inform policy and training.

## Methods

### Recruitment and sampling

Qualitative research offers an insight into the experiences and perceptions of the cohort of interest in situations where little is known about the research topic. Qualitative data were collected via individual 30-60 minute semi-structured interviews with GP supervisors, GP registrars, prevocational trainees, medical students and practice managers (PMs) situated in teaching-accredited practices, to investigate their perceptions of shared learning.

Recruitment and data collection occurred between September, 2011 and March, 2012 via an email invitation from the regional training provider (see footnote 1) outlining the research aims, project requirements and participants’ rights. Purposive sampling was used to seek a range of participants from multiple practices (n=9) that had more than one level of learner in order to obtain a cross-section of views (Table 1). Thirty-nine percent of GP supervisors, 44% of registrars, 40% of prevocational trainees, 73% of medical students and 44% of practice managers who were approached consented to be interviewed. Informed consent was obtained from each volunteer. Sampling continued until data saturation was reached [15], ie. no new data emerged. The study area included a ~500 km coastal strip of NSW, Australia, that stretches from ~400 km north of Sydney (the Port Macquarie region) to ~100 km south of Brisbane (the Tweed Heads region).

**Table 1:** Practice demographics

**Practice**	**No. GP teachers in**	**Levels of learner in**
	**the practice**	**the practice**
1	2	GPT1 registrars
		GPT2 registrars
		GPT3 registrars
		Medical students
2	3	GPT2 registrars
		Prevocational trainees
		Medical students
3	3	GPT2 registrars
		GPT3 registrars
		Medical students
4	9	GPT2 registrars
		Prevocational trainees
		Medical students
5	4	GPT1 registrars
		GPT2 registrars
		GPT3 registrars
6	3	GPT1 registrars
		GPT2 registrars
		GPT3 registrars
		Medical students
7	1	GPT2 registrars
		GPT3 registrars
		Extended skills registrars
8	3	GPT2 registrars
		GPT3 registrars
		Medical students
9	2	GPT1 registrars
		GPT2 registrars
		Extended skills registrars
		Prevocational trainees

### Ethical approval

Ethics approval was obtained from Southern Cross University’s Human Research Ethics Committee (ECN-11-187). To maintain anonymity, all participants are subsequently referred to by a code that indicates gender, the stakeholder group they represent, and a number. For example, FGPR83 is a female GP registrar, and her code is 83. Male gender is indicated by M.

### Instruments

The interview questions were developed by the researchers, and reviewed by an advisory group that included medical educators, university faculty, an education consultant, and a GP registrar. The interview topics are displayed in Appendix 1.

### Data collection and analysis

Interviews conducted by phone (58%) or in person (42%) by TM, CA and PS were recorded and transcribed by an independent transcriber. CA, TM and PS independently conducted a thematic analysis of the data as outlined in Braun and Clarke [16], using the software package NVivo9 [17]:

1. Becoming familiar with the data through reading and re-reading the transcript and actively searching for patterns and meaning.

2. Production of initial codes which identify a basic element of raw data to which meaning can be attached.

3. Searching for themes and grouping codes into broader themes.

4. Reviewing and refining themes.

5. Identifying the essence of what each theme is about, and identifying sub-themes within the themes.

6. Producing a report with data extracts that capture the essence of the findings.

Consensus was achieved through discussion. Independent analysis of the data (investigator triangulation) provides credibility [18]. Additionally, quotes from participants have been included as described by Braun and Clarke [16]; in this way the findings demonstrably reflect the participants’ voices [18].

## Results

Thirty-three participants comprising 11 GP supervisors, eight registrars, two prevocational trainees, eight medical students and four practice managers located in nine general practices in northern NSW, Australia were interviewed. Various shared learning models were being utilised (Table 2).

**Table 2:** Types of shared learning activities

**Type of activity**	**No. of practices**
Group education sessions run by GP	7
Education sessions shared between geographically co-located practices	2
Group education sessions followed by:	
• Structured 1-1 with junior learners	4
• Informal 1-1 with learners	3
Registrar and medical student observe GP consultations	2
Integrated ward rounds	1

Two overarching themes were found: Benefits and Risks (Table 3). Subthemes included Quality of Teaching; Effectiveness of Learning; Group Dynamics, Interpersonal and Personal Issues; Financially Rewarding; Workplace Satisfaction; Supervisor Learns; Maintaining Teaching Quality; Financial Efficiencies; Increased Sustainability; a Quality Improvement Process; and May Increase Practice Workload.

**Table 3:** Summary of participants’ perceptions of the benefits and risks of shared learning models

**For**	**Subtheme**	**Benefits**	**Risks**
**Learners**	Quality of teaching	• Everyone challenges each other, encouraging debate and discussion	• Junior learners require more 1:1 teaching
		• Extra questions are asked that the individual learner did not think of	• Attempting to meet needs of multiple levels of learners runs the risk of not meeting anyone’s learning needs
		• The group can learn from the expertise, knowledge, skills of others in the group	• Level may be too low or too advanced
		• Learners discover different approaches to same problem	• Less personalised teaching and fewer opportunities to address individual learning needs when compared to 1:1 teaching
		• Provides early exposure to advanced skills for junior learners	
		• Learning increases for all levels	
		• Resources can be more easily shared	
	Effectiveness of learning	• Easier/safer to ask questions	• Different clinical approaches may confuse learners
		• A difficult topic explained to others can aid learners’ understanding	• Shared learning models are unsuitable when learners:
		• Shared learning is active learning because it requires more preparation and interaction compared to 1:1	1. Require remediation
			2. Have a specific deficit that needs to be addressed
			3. Are given personalised feedback
		• Shared learning sessions are more likely to be structured and planned and may lead to better learning outcomes	4. Are observed by their supervisor
			5. Have immediate learning needs
			• Shared learning models are less effective for learning communication and procedural skills
	Group dynamics, interpersonal and personal issues	• Takes pressure off the individual learners to answer all the questions	• Learners less comfortable asking questions in group situation than 1:1 teaching
		• More collegial, builds relationships, is enjoyable	• One person may hijack the meeting
		• Stimulating/supportive environment	• Shy learners may not learn as much
		• Learning in a group can spur everyone onto to greater efforts	• Junior leaners fear imposing on senior learners
		• Reduced feelings of isolation	• Shared learning is unsuitable when learners have sensitive or embarrassing issues to discuss
		• Being able to benchmark against peers improves self-confidence	
		• Confidence to acknowledge lack of knowledge or skills is less threatening if other learners demonstrate the same	
		• Being able to debrief and share difficult situations with other learners improves self-confidence	
**GP supervisor**	Financially rewarding	• More financially rewarding due to increased clinical time and higher payment per hour of teaching	
	Workplace satisfaction	• Increased engagement and less repetition in teaching	• More stressful than one-to-one
		• Reduced workload due to time efficiency	• May require more planning
		• Less chance of burnout or stress	
	Supervisor learns	• Introduction to new techniques, information and theories by learners, often from those who recently came from big hospitals	
	Maintaining teaching quality		• Different people require different teaching styles
			• Teaching quality depends on GP’s practice, personality, experience and teaching style
			• More difficult to address the needs of all levels of learners
**Practice**	Financial efficiencies	• More financially beneficial due to increased time to generate revenue	
	Increased sustainability	• Increased likelihood of sustainable practice in terms of financial viability and sustainable employability of general practitioners	
		• Increased vitality in the practice	
	A quality improvement process	• Provides a forum to standardise and improve patient management	
	May increase practice workload		• May require more planning
			• Lack of standardisation of teaching between practices

### Benefits of shared learning

Learners perceived shared learning provided an environment that was more conducive to discussion and debate ‘*because everyone challenges everyone, everyone keeps everyone honest’* (MMS25) and *‘in VI it’s probably easier to question and go, “well what about that” versus one-on-one [which is] more mentor-student role’* (MGPR92).

Some learners suggested that the learning experience was broader because *‘other people will ask interesting questions that you might not have thought of’* (FGPR37), ‘*everyone’s got their strengths’* (MGPR92), and everyone learnt from the combined knowledge, experience and approaches of the wider group. For example,

*‘different people have different knowledge bases… …not only do you gain a better breadth of experience because more people are putting their opinions in, but you can also learn from other people who aren’t necessarily more senior to you’* (FGPR34).

Supervisors concurred that ‘*[students and trainees] have often had other careers, [and] bring a whole lot of expertise from other areas and everyone just has different interpersonal skills or interests so you just get different viewpoints on things, so I think it’s more educational basically”* (MGP18). Supervisors also felt that ‘*a VI teaching environment would have a bigger impact for change. I just think it’s the hierarchical nature of it that across the board everybody is trying to show the other guys that they are as good or better’,* and that spurs everyone on to greater efforts. (MGP11). Additionally, shared learning *‘gave the opportunity for the advanced person to learn some advanced skills and also the junior person…to at least get some exposure to them, so everybody had a win’* (MGP11).

Shared learning can be more effective because hearing a difficult topic explained to others can aid learners’ understanding. The learning is also more active as shared learning sessions encouraged more preparation and interaction*,* for example, one registrar suggested *‘with VI [you are] more likely to do readings perhaps versus one-on-one [where] it’s easy to sit back and make the GP the guru and just absorb knowledge that way versus to contribute’* (MGPR92).

Learners suggested that shared learning takes the pressure off the individual as *‘the load’s not entirely on me alone to answer all the questions’* (FGPR34), and ‘*vertical teaching is great because it can be a bit isolating as a young person in a community’* (FMS33), and learning in a group was more enjoyable, stimulating, and collegial: ‘*I actually like the VI sessions… It’s nice to spend some time with people in a non-threatening environment that aren’t patients, it’s good…it’s social*…*it makes me a bit more excited about learning than it would just the one-on-one sessions’* (FGPR83).

Learners found that benchmarking themselves against other learners built confidence, and was reassuring, suggesting ‘*it helps hearing what other registrars know and don’t know, rather than going into sessions where you’re always with a supervisor who seems to know everything…being able to admit that you don’t know stuff is helpful in a learning situation when there’s more than one person saying, “I don’t know that either” ‘* and ‘*checking that other registrars generally follow the same patterns you do is confidence building*’ (FGPR83). Another described discussing with the group a consultation that had gone wrong, and *‘I felt way better… hearing other people’s stories where they’d stuffed up and had bad things happen’* (MGPR92).

Shared group learning sessions can also improve patient management. For example,

‘*the GP always started with me, in terms of asking questions and then moved … up the line in terms of asking about patient management…to talk about what they could have done better. It was really useful. It was a retrospective look at the patient list, how things might have been improved’* (FMS4)

Supervisors and practice managers perceived that shared learning sessions reduced workload through time efficiencies, and were more financially rewarding for the practice, reducing the impact on clinical time and billings and increasing the hourly payment for teaching.

*‘in terms of convenience, logistics and actually being viable financially, if you get paid for [each] learner, having more than one in the room at a time works. If you’ve got to do it one-to-one, you lose money hand over fist’* (FGP42)

‘*if I teach two registrars and one [PT], essentially I am doing three hours of teaching in one hour. That’s a very efficient and effective way of delivering teaching. That’s the single biggest advantage, it’s time effective’* (MGP16)

Shared learning models also reduced supervisor burnout, as ‘*the supervisors don’t have to do multiple sessions. We’ll have three registrars next year. [Instead of] having to do the same stuff three times … doing it all at once definitely lessens the workload…a little bit friendlier for burnout…if you get it all done at once it frees up everyone for doing things, less time out of billing [and] clinical working time’* (MPM51).

Supervisors learn from the group, and the practice benefits from exposure to the newest techniques and information, for example, ‘*the smaller the town the more beneficial [shared learning] would be; [recently a student] introduced the idea of a CHAD score, as embarrassing as it may seem, and we all sat there looking at them going, ‘never heard of it’…and our practice is now very up-to-date with it and uses it constantly…I find the students particularly, and the registrars, because they’ve often just recently come from bigger hospitals, bring with them a wealth of information’* (MGP18).

Teaching multiple learners together also ‘*saves rehashing the same sort of stuff*’ (MPM51), and supervisors suggested ‘*it’s probably stress lowering because they’re quite enjoyable sessions’* (MGP18), and ‘*I think there are strong positives in having VI teaching specifically…It gives a vitality to practice, it looks a lot more alive and fresh and new’* (MGP18). The joint educational session also potentially *‘impacts on patient care indirectly. I know if I’m teaching to all levels from the same gimble…that they’re all on the same page as to my approach or philosophy on treating a certain disease’* (MGP11).

### Risks and issues

Some learners perceived that shared learning could adversely impact on learning outcomes because a session can be hijacked *‘just because someone interjects a lot’* (FMS33), that *‘there’s no one teaching style that’s good for everybody’* (FGPR34), and the attempt to meet the needs of all levels means ‘*the majority not having whatever it is at their level*’ (MMS12). For example,

*‘the benefits of having one-on-one teaching is it’s specifically designed for my level of knowledge …the negatives [of shared learning are that] I have to sit through all the basic stuff for the people who are more junior to me…before getting to the level of stuff that I need to know’* (FGPR34)

Learners also suggested that *‘you’re not always as comfortable to ask questions that you might’ve been in an individual session.. [there’s] some stuff that you don’t want to bring up in a group’* (FGPR12), and described *‘a lack of confidence. Feeling stupid, judged if you ask a question, that’s the main obstacle’* (FGPR35).

Learners suggested the variability of teaching skills also impacted on the quality of the experience, for example, ‘*it really is very variable depending on what the teaching quality is like in the different practices’* (FGPR83), and ‘*it depended a lot on the teacher because they all had very different styles’* (FPT4).

A number of learners felt that ‘*a mixture of both [VI and non-VI] … has its benefits’* (FGPR34) providing opportunities for both collegial interaction and personalised learning. From the perspectives of supervisors, ‘*you have to be able to pitch it at all levels. It’s a lot more challenging to get it right…VI teaching is a wee bit more stressful as you have to consider all the players, all their needs, all their expectations, but it’s balanced out by the positives*’ (MGP11). There was also the suggestion that ‘*the discussions about the detail could sometimes confuse the learner more because often there can be different approaches … it is harder on the learner to actually make up their mind what they will do*’ (FGP66).

### Is shared learning always appropriate?

Learners felt that one-to-one teaching was more suitable when:

•they were pressed for time or had very specific questions they wanted answered to meet their immediate learning needs, ‘*you can talk about your immediate concerns*’ (FGPR37), ‘*if you’ve got very specific questions*’ (FPT6),

•there were sensitive situations, ‘*if I’ve got more sensitive problems or I’m concerned about something and I’d rather just [do non-VI]* (MGP92); ‘*at the end of the day .. my [supervisor would] look through every single case that I’d done that day and talk about what could’ve been done differently…which was great for my personal learning but you feel quite scrutinised and I think scrutiny in front of more than one person is a bit yucky’* (FPT6),

•junior learners did not want to impede the learning of seniors, eg. ‘*if it’s allocated registrar training time, I don’t want to say “oh, I don’t understand that”’* (MMS25),

•juniors felt shy about speaking up in groups, ‘*I’ve often found, in a setting with interns and registrars, medical students feel a bit awkward speaking up because they feel the lack of knowledge, they feel embarrassed. I often feel embarrassed about asking questions and putting forward ideas because you have this feeling that you don’t know enough to do it. So I find one-on-one particularly important’* (MMS12).

Supervisors felt juniors needed more one-to-one teaching and one-to-one was better when:

•‘*you have to [have] remedial teaching for those trainees and obviously that’s going to be a one-to-one situation’* (MGP11),

•‘*you have to really specifically target a knowledge deficiency … there’s going to be some pain in addressing that in a VI format’* (MGP11),

•Teaching communication and procedural skills, as this is learnt better by watching and mirroring than shared teaching time, ‘*I did a toenail excision … the other day… unless I’m doing it with my hands, it’s no good, so let’s say that another reg was doing it, it’s not that tactile an experience and so I think procedural stuff is probably better one-on-one’* (MGPR92), and ‘*if you’ve got a registrar who’s struggling with communication skills, then really we might want to be focussing on just those issues and having other people just takes away from that focus and so it’s probably better to have a one-on-on*e’ (MGP7),

•‘*Sitting in on the registrar and watching them*…*Giving feedback’* (FGP27),

•Managing really shy learners, ‘*if they’re not asking questions they’re most likely not learning what they could. I guess it depends … how they learn best, so if we do get a registrar that isn’t able to learn very well in a group environment then it’s either look at our supervisors or make way for some one-on-one training’* (MPM51).

## Discussion

This study identified various benefits and risks of shared learning models, and identified under which circumstances it is appropriate to use shared learning and when it is more appropriate to use one-to-one teaching in general practice. To our knowledge appropriate use of shared learning has not been previously discussed in the literature.

Participants clearly saw the benefits of shared learning for group dynamics and interpersonal relationships within the practice. Benefits previously discussed in the literature that were empirically confirmed by a larger sample and wider range of learners in this study included improved morale, collegiality, patient care, benchmarking of learning, and learning from others’ mistakes [9,13,14]. Benefits previously hypothesised in the literature for GP supervisors that were confirmed by our participants included a reduction in workload [12] and an opportunity for supervisors to keep up to date [13] through new information brought to the practice by learners. While Laurence et al. [19] previously determined that a net financial gain was achieved when GPs supervised more than one registrar at the same level concurrently, our supervisors suggested that cost efficiencies could be obtained through shared learning sessions when supervising multiple levels of learner concurrently. The reduction in burnout for supervisors through avoidance of repetitively teaching the same topics separately to multiple learners is a new finding.

Previously hypothesised risks and issues with shared learning models confirmed by our participants included variable learning needs of learners, and variable teaching quality [13,14]. An additional risk proposed by our participants was that some learners had reduced confidence to ask questions in a group setting. Some GPs also experienced more stress during facilitation of shared learning sessions, finding it harder to address the needs of all levels of learners, although they felt the benefits outweighed the disadvantages. Shared learning models were considered unsuitable when learners needed to: receive remediation, address a specific deficit or immediate learning needs, learn communication or procedural skills, be given personalised feedback or be observed by their supervisor during consultations. They were also not appropriate when learners had time pressures, were really shy or had embarrassing or sensitive issues that they wished to discuss.

The major implication of these findings is that shared learning appears to offer an opportunity to increase training capacity in general practice, both directly through time and cost efficiencies, and more indirectly through increased supervisor satisfaction and reduced burnout from repetitive teaching. Providing training for supervisors in small group facilitation skills may offset the risk of increased stress related to juggling the demands of multi-level teaching, and improve learner satisfaction with group teaching also.

These findings also suggest that supervisors who are solely using one-to-one teaching are missing opportunities to reduce social isolation and workload, and improve their own learning and that of learners and other staff in the practice. For GPs, a clear benefit was the introduction to new techniques, information and theories by learners, often from those who recently came from big hospitals. This suggests that improved communication between larger hospitals and smaller general practices is desirable to ensure GP supervisors stay up-to-date. Learners can be an important ‘bridge’ between the two. Given learners are often unaware of, or lack confidence in, their knowledge they should be encouraged to share knowledge with their senior colleagues. This builds the knowledge base of the supervisor *and* the learner’s confidence to interact in the group.

Conversely, those considering using shared learning models exclusively may want to take into consideration that some things are not taught appropriately or well in a shared learning environment. Providing a mixture of both shared learning sessions and one-to-one teaching is beneficial because it would provide opportunities for both collegial interaction and cross-pollination of ideas as well as personalised learning, allowing stakeholders to reap the benefits of both models while minimising the disadvantages of either, thereby maximising the learning outcomes for everyone. On the whole, learners appeared to prefer a mixture of the two models.

Based on Wenger et al. [11] definition of communities of practice, the shared learning models described by some of our participants fitted the description of, and provided many of the beneficial outcomes attributed to communities of practice, such as helping each other with difficult cases and developing new perspectives through collective reflection [11]. At their best shared learning communities can be inspiring, fun, build confidence and social capital, and facilitate learning [20]. It may be time to move from the one-directional knowledge transmission from master to apprentice model, and informal, tacit community of practice models currently established in some general practices, to formal recognition of the general practice as a community of practice with an explicit goal of multi-directional knowledge transmission and skill development. This community of practice may be within one general practice or across multiple geographically contiguous practices. Factors that contribute to a successful community of practice model in the general practice setting should be explored.

One of the strengths of the study is that it sought the views of all key stakeholders in relation to shared learning including supervisors, practice managers and all levels of learner, which has not been done previously. There were several limitations. Any self-report research is subject to potential biases. For example, participants may adhere to a cultural model because they feel it has more prestige, and they wish to appear to conform to what they see as the norm, or most socially acceptable [21]. Having said that, shared learning is not the norm in general practice, and participants appeared willing to provide their views on both the negatives and positives of shared learning. Another limitation was that the interviewers were employees of the local GP training provider, which may have influenced the interviews and objectivity of the data analyses. Additionally, the general practices that took part are from rural Australia and are more likely to be early adopters of shared learning models, and the results are not generalisable. However, while the number of participants in the study (particularly prevocational trainees) was comparatively small, there is some support from the literature to indicate that the findings are reliable.

## Conclusions

Shared learning models can be a type of informal community of practice that at their best make learning enjoyable, build social capital, and improve learning outcomes through engendering active learning and tapping into the knowledge and skill of the whole group. However, our participants suggest that one-to-one teaching is more suitable in some situations. A mixture of shared learning and one-to-one teaching would allow stakeholders to reap the benefits of each model while minimising the risks, thereby maximising the learning outcomes for all.

To further build the evidence, a national quantitative study is required to ask all key stakeholders what they see as the benefit and risks of shared learning and what determines the elements of an effective model. Additionally, future research could focus on using objective methods to measure the impact of shared learning models.

## Endnotes

^a^Australian general practice training is funded by the federal government through a body called General Practice Education and Training (GPET) (http://www.gpet.com.au/). GPET funds 17 Regional Training Providers (RTPs) to manage the placements of GP registrars (vocational trainees) in private general practices in their region. RTPs accredit practices and supervisors to take on registrar training, and also provide some mandatory group training sessions for registrars during their terms. The remainder of the training is provided by GP supervisors through a range of supervisory mechanisms such as one-to-one teaching, group learning sessions, and parallel consulting, as well as informal ‘corridor’ teaching. GP registrars undertake either a three or four year program, depending on which of the two colleges of general practice they are training towards. The practices that responded to this study are all training registrars towards Fellowship of the Royal Australian College of General Practitioners, which includes a hospital year (not relevant to this study), as well as three six-month training terms referred to as their GPT1, GPT2 and GPT3 terms, which are generally undertaken in private general practices. A fourth (extended skills) term is often undertaken in the general practice setting as well. GP registrars in private practice generally receive a proportion of their billings commensurate with their level of experience as income. More junior registrars (those in their first two terms) are protected by an agreement called the National Minimum Terms and Conditions, which mandates a minimum salary and leave provisions.

Junior doctors in hospitals who have not yet decided on their career path have the option of doing a 10-13 week placement in a primary care setting, most often in private general practice, under the Prevocational General Practice Placements Program. RTPs manage this program. Prevocational trainees conduct patient consultations under the supervision of a GP supervisor and also require additional teaching time.

In addition, medical students do placements in general practice during their medical degree. These placements are managed by universities or Rural Clinical Schools (RCSs), which are entities developed to oversee placements and training for medical students and allied health students in rural settings. The length of the general practice placement varies widely between universities and RCSs and stage of the degree, from as little as one week to as much as 12 months.

Practices are paid to take all levels of learner. The practice receives a Practice Incentive Payment for each session the medical student spends in the practice, and this payment comes directly to the practice from the commonwealth government via the Medicare system. There are also financial subsidies provided for practices that take GP registrars and prevocational trainees, and supervisors are reimbursed for a set number of teaching hours each week during the first two training terms for GP registrars, and for all prevocational trainees. Additionally the practices earn income from the billings of the registrars and prevocational trainees.

## Appendix 1: interview questions

### GP supervisors

For the purpose of this research, **teaching** is defined as structured education sessions such as lectures, tutorials, case discussions, journal clubs, ward rounds, or wave or parallel consulting with medical students. This definition of teaching excludes corridor or phone call teaching.

For the purposes of this study ‘**Vertical Integration (VI)’** refers to the delivery of education sessions by the teacher simultaneously to multiple levels of learners. A VI teacher is someone who teaches to multiple levels of learners simultaneously.

1. Why did you choose to do this style of teaching?

2. How do you approach your VI teaching sessions?

3. Could you describe the experience of being a teacher of VI education sessions in this general practice?

4. Do you feel that the learners change their knowledge, skills or attitudes as a result of the VI teaching you have provided? If so could you give any examples?

5. How did you feel having other learners present influenced the experience?

6. What do you see at the impacts of VI teaching on you, both positive and negative?

7. What are the impacts, both positive and negative of VI teaching on the business?

Repeat questions 1-7 focusing on non-VI teaching style this time.

8. As a teaching modality do you think that VI or non-VI teaching is more effective, and why?

9. Is there anything else you would like to add?

**Learners** (GP registrars, Prevocational Trainees, medical students)

For the purpose of this research, **teaching** is defined as structured education sessions such as lectures, tutorials, case discussions, journal clubs, ward rounds, or wave or parallel consulting with medical students. This definition of teaching excludes corridor or phone call teaching.

For the purposes of this study **‘Vertical Integration (VI)’** refers to the delivery of education sessions by the teacher to multiple levels of learners simultaneously (for eg. Medical student and GPR in the same session).

The following questions relate to your experiences in the General Practice you are currently stationed at:

1. What type of VI teaching do you receive?

2. How frequently does VI teaching occur in this practice?

Thinking about the VI sessions run by the GPs in this practice, please answer the following questions:

3. Could you describe the experience of being a learner in the education sessions you have received at this practice?

4. Did you feel that your knowledge, skills or attitudes changed as a result of the teaching sessions you attended? If so, could you describe in what ways?

5. Did you feel having other learners present influenced the experience?

Repeat questions 3-5 this time with a focus on non-VI teaching to examine the learner’s non-VI experiences and perceptions.

6. As a teaching modality, do you think that VI or non-VI teaching is more effective and why?

7. Is there anything else you would like to add about your experience of being a learner in the general practice setting?

### Practice managers

For the purpose of this research, **teaching** is defined as structured education sessions such as lectures, tutorials, case discussions, journal clubs, ward rounds, or wave or parallel consulting with medical students. This definition of teaching excludes corridor or phone call teaching.

For the purposes of this study **‘Vertical Integrated (VI)’** Practice refers to Practices in which there is delivery of education sessions by the teacher to multiple levels of learners simultaneously (for eg. Medical student and GPR in the same session).

1. What is the impact (both positive and negative) of VI (and/or non VI-style) teaching on the Practice and on you personally?

2. Is there anything else you would like to add?

## Competing interests

The authors declare that they have no competing interests.

## Authors’ contributions

All authors contributed to the conception of the study and participated in the critical revision of the manuscript and read and approved the final version. CA, PS and TM collected and analysed data. TM drafted the manuscript. All authors read and approved the final manuscript.

## Authors’ information

CMA (MBBS, FRACGP) is the Director of Training at North Coast GP Training. TFvdM (PhD, BSc(Hons), RN) is the Research Director at NCGPT and an academic at Southern Cross University. PLS (MBBS, FRACGP, Dip Pub Hlth) is a GP and Medical Educator with NCGPT. JAB (PhD, RN) is an academic at Southern Cross University. SWP (PhD) is a researcher in the University Centre for Rural Health, University of Sydney.

## Pre-publication history

The pre-publication history for this paper can be accessed here:

http://www.biomedcentral.com/1471-2296/14/144/prepub
